# Successful resection of ampullary neuroendocrine tumors using clip traction-assisted hybrid endoscopic submucosal dissection

**DOI:** 10.1055/a-2604-8062

**Published:** 2025-06-26

**Authors:** Ke Jiang, Justin Ryan Lay Tan, Weigang Gu, Qifeng Lou, Hangbin Jin, Jianfeng Yang, Xiaofeng Zhang

**Affiliations:** 1 70571 The Fourth School of Clinical Medicine, Zhejiang Chinese Medical University, Hangzhou First Peopleʼs Hospital, Hangzhou, China; 2 106055 Section of Gastroenterology, Chinese General Hospital and Medical Centre, Manila, Philippines; 3 Department of Gastroenterology, Affiliated Hangzhou First Peopleʼs Hospital, Westlake University School of Medicine, Hangzhou, China

A 63-year-old man was referred due to a 1-month history of abdominal pain. Endoscopic ultrasound (EUS) and endoscopic retrograde cholangiopancreatography (ERCP) showed a 2.1 × 2.3 cm duodenal papilla submucosal tumor (SMT) with distinct borders ( Fig. 1 ). Endoscopic submucosal dissection (ESD) was initially planned. A submucosal injection of diluted methylene blue was performed, followed by a mucosal incision using a GoldKnife (Microtech Nanjing). This was followed by a layered multi-knife dissection (Gold Knife and IT Knife Nano) performed with clip traction assistance ( Fig. 2 **a, b** ). Due to anatomical challenges and bleeding, hybrid-ESD was adopted ( Fig. 2 **c, d** ). Placement of pancreatic and biliary stents for endoscopic retrograde pancreatic drainage (ERPD) and endoscopic retrograde biliary drainage (ERBD) and the post-resection defect were closed with a total of seven hemoclips. A nasobiliary tube was inserted to monitor the bleeding ( Fig. 3 , Video 1 ). The patient was discharged 7 days post-procedure.

**Fig. 1:**
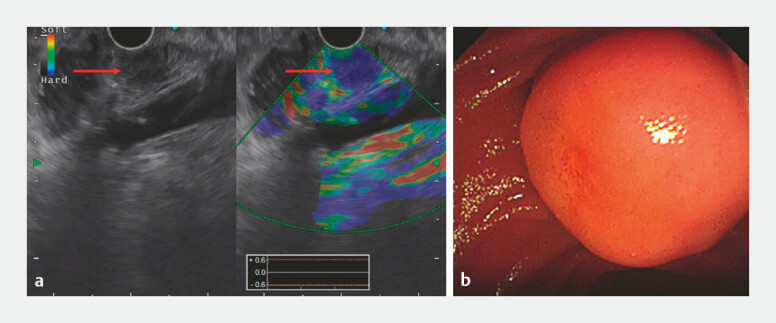
EUS ( **a** ) and endoscopic image ( **b** ) showing the duodenal papilla submucosal tumor. Abbreviation: EUS, endoscopic ultrasound.

**Fig. 2:**
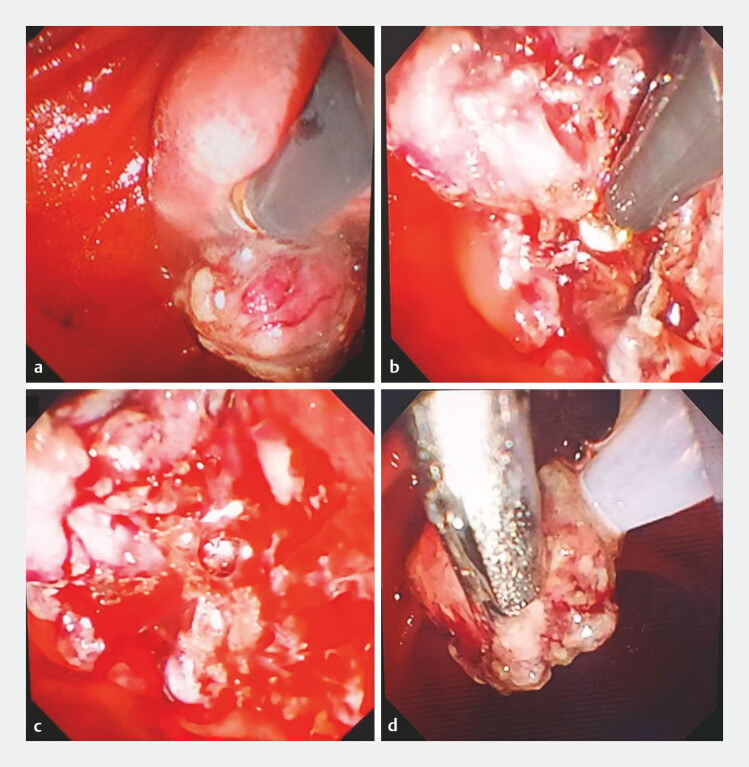
Surgical process images of hybrid ESD and EMR assisted by clip traction: **a** Using a Goldknife for submucosal dissection; **b** Using an IT Knife Nano for submucosal dissection; **c** Intraoperative bleeding resulted in blurred field of view, making it impossible to proceed with ESD for complete resection; **d** EMR of the partially dissected lesion. Abbreviations: ESD, Endoscopic submucosal dissection; EMR.

**Fig. 3:**
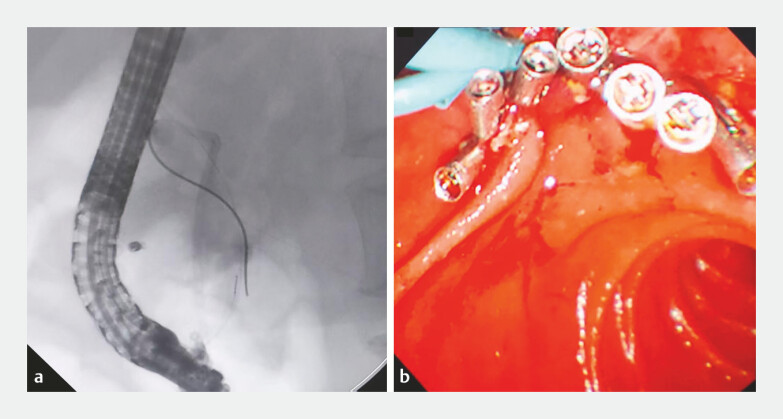
**a** ERCP was performed to insert a 7Fr single-pigtail biliary stent (3 cm) and a 7Fr single-pigtail pancreatic stent (7 cm) to prevent biliary stricture and pancreatitis, respectively; **b** A total of seven hemoclips (Micro-Tech) were deployed to close the post-ESD defect and a nasobiliary tube was inserted to monitor the bleeding. Abbreviation: ESD, endoscopic submucosal dissection.

Successfully resected neuroendocrine tumors in the ampulla using a combination of ESD and EMR with a clip-assisted traction.Video 1

The combined maximum diameter of the two specimens measured 1.9 cm ( Fig. 4 **a, b** ). Histopathology and immunohistochemistry showed a well-differentiated neuroendocrine tumor (NET), classified as G1 ( Fig. 4 **c, d** ), with tumor-free resection margins. Follow-up ERCP after 1 month demonstrated complete healing of the excision site, and random biopsy results were normal ( Fig. 5 , Video 1 ). The patient remained asymptomatic with no evidence of recurrence at the 12-month follow-up.

**Fig. 4:**
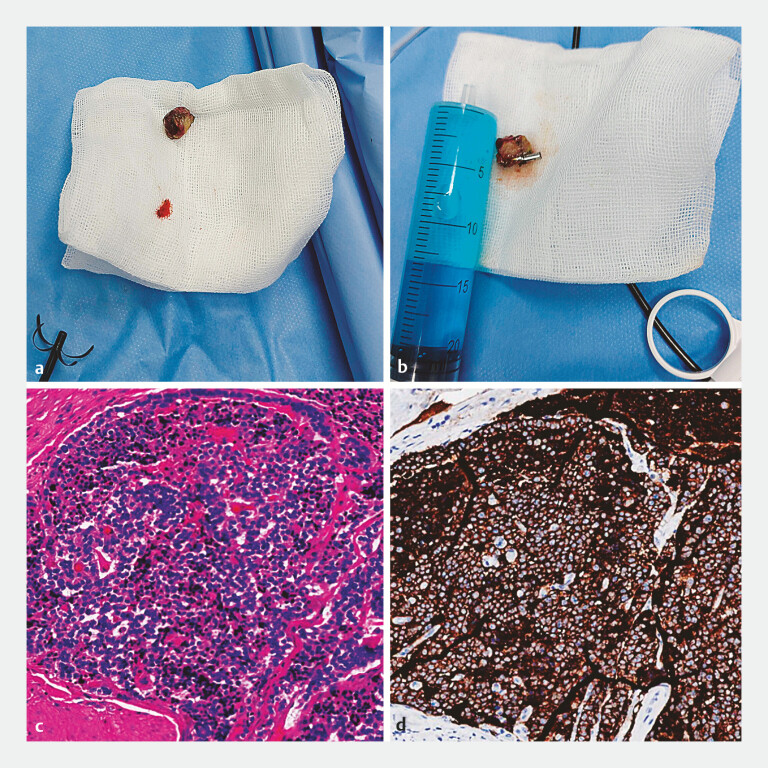
The combined widest diameter of the two specimens was 1.9 cm ( **a, b** ); Histopathology and immunohistochemistry showed a well-differentiated NET, classified as G1 ( **c, d** ). Abbreviation: NET, neuroendocrine tumor.

**Fig. 5:**
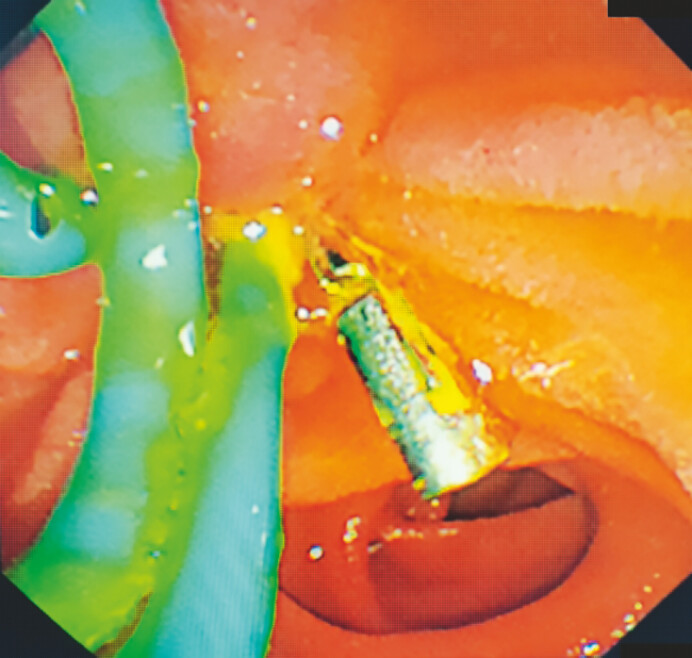
Image from follow-up duodenoscopy 1 month later showing complete healing of the resection site and no evidence of recurrence.

The unique anatomical features of the duodenum pose certain challenges to the implementation of ESD 1 2 3 4 . In this context, we highlight the utility of a single-clip-assisted traction technique combined with a novel hybrid-ESD approach for treating ampullary lesions. Our experience demonstrates that this technology effectively enhances surgical field exposure, thereby improving dissection efficiency and the overall resection rate. Additionally, it reduces risks such as bleeding and perforation.

Endoscopy_UCTN_Code_TTT_1AO_2AG_3AD

## Notes

Endoscopy E-Videos https://eref.thieme.de/e-videos
*E-Videos* is an open access online section of the journal *Endoscopy* , reporting on interesting cases and new techniques in gastroenterological endoscopy. All papers include a high-quality video and are published with a Creative Commons CC-BY license. Endoscopy E-Videos qualify for HINARI discounts and waivers and eligibility is automatically checked during the submission process. We grant 100% waivers to articles whose corresponding authors are based in Group A countries and 50% waivers to those who are based in Group B countries as classified by Research4Life (see: https://www.research4life.org/access/eligibility/ ). This section has its own submission website at https://mc.manuscriptcentral.com/e-videos .
